# Editorial: Proceedings of FSTP3 Congress–A Sustainable Durum Wheat Chain for Food Security and Healthy Lives

**DOI:** 10.3389/fpls.2021.675510

**Published:** 2021-04-09

**Authors:** Roberto Tuberosa, Luigi Cattivelli, Aldo Ceriotti, Agata Gadaleta, Brian L. Beres, Curtis J. Pozniak

**Affiliations:** ^1^Department of Agricultural and Food Sciences (DISTAL), University of Bologna, Bologna, Italy; ^2^Council for Agricultural Research and Economics, Research Centre for Genomics and Bioinformatics, Fiorenzuola d'Arda, Italy; ^3^Institute of Agricultural Biology and Biotechnology, National Research Council (CNR), Rome, Italy; ^4^Department of Agricultural and Environmental Science (DISAAT), University of Bari “Aldo Moro”, Bari, Italy; ^5^Lethbridge Research and Development Centre, Lethbridge, AB, Canada; ^6^Crop Development Centre, University of Saskatchewan, Saskatoon, SK, Canada

**Keywords:** durum wheat, germplasm collections, drought resistance, disease resistance, genome-wide association study (GWAS), quantitative trait locus (QTL), yield sustainability, grain quality

This Special Issue of Frontiers in Plant Science collects 14 manuscripts presented at the Congress ‘From Seed to Pasta 3' (www.fromseedtopasta.com/). The papers highlight some of the most recent achievements in durum wheat (*Triticum turgidum* ssp. *durum*) research, toward a sustainable durum wheat chain able to enhance food security and healthier grain production. A timely update that gauges the impressive progress achieved since the publication of the first Special Issue on durum wheat genomics (Tuberosa and Pozniak, 2014).

The studies address issues relevant for climate-resilient production of durum wheat, particularly in North African countries where durum wheat is a staple to more than 100 million people. Beres et al. describe how meeting this challenge requires matching new cultivars with the best modern management practices. Further, the authors argue that durum production faces stresses that curtail yields and quality specifications desired by export market end-users. Thus, successful biotic and abiotic threat mitigation are ideal case studies in Genotype (G) × Environment (E) × Management (M) interactions where superior cultivars (G) are grown in at-risk regions (E) and require unique approaches to management (M) for sustainable durum production that suit specific agro-ecozones and close the gap between genetic potential and on-farm achieved yield.

From a breeding standpoint, a pivotal issue remains having access to and leveraging beneficial allelic diversity to support genetic studies (Pozniak et al., 2012) and to enhance the performance of elite cultivars (Salvi and Tuberosa, 2015; Khalid et al., 2019; Brinton et al., 2020). A stepping stone to address this issue is the assembly of the Global Durum wheat Panel (GDP), a collection of 1,018 lines designed to identify beneficial alleles for durum wheat improvement (Mazzucotelli et al.). The GDP captures the majority of the diversity available in modern durum wheat germplasm and landraces along with a selection of Emmer and primitive tetraploid wheats to maximize biodiversity. Public seed availability and complete genetic characterization of the GDP provides a unique resource to identify and exchange beneficial genes and alleles to enhance durum wheat breeding world-wide. The GDP is now complemented and extended by the Tetraploid wheat Global Panel (TGC), suitable for a more accurate haplotyping owing to its lower decay of linkage disequilibrium (Maccaferri et al., 2019) and for rare allele identification (Figure 1).

**Figure 1:**
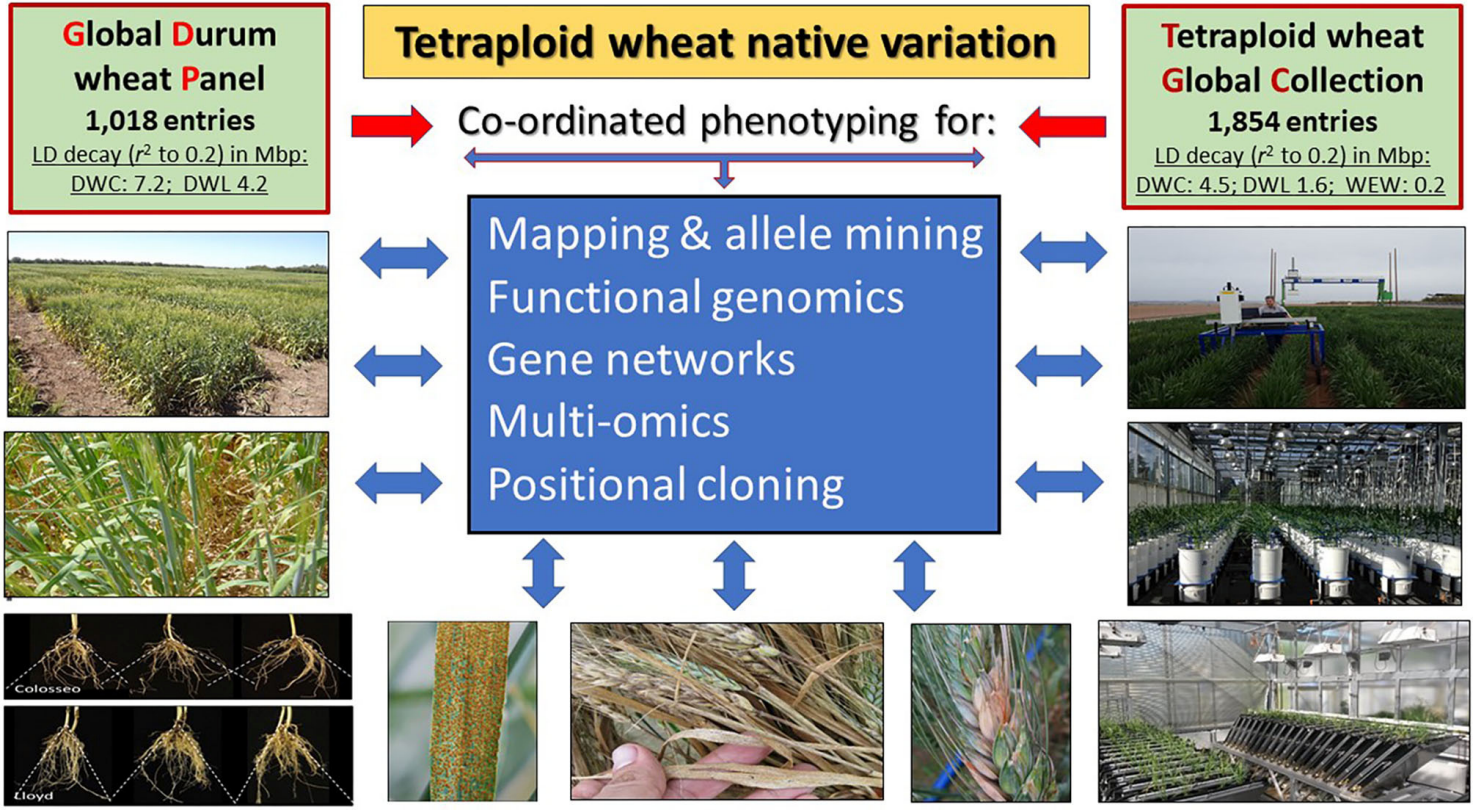
The Global Durum wheat Panel (GDP; Mazzucotelli et al.) and the Tetraploid wheat Global Collection (TGC; Maccaferri et al., 2019) are instrumental to mine the vast biodiversity present in the A and B genomes of *Triticum* species. The lower linkage disequilibrium (LD) decay of the TGC suggests its suitability for cloning QTLs while the GDP is more suitable for breeding purposes. DWC, Durum Wheat Cultivars; DWL, Durum Wheat Landraces; WEW, Wild Emmer Wheat.

A multi-trait, multi-environment genomic prediction model based on genomic best linear unbiased predictor (GBLUP) coupled with a deep-learning method (DLM) was adopted by Montesinos-López et al. to predict grain yield, days to heading and plant height of 270 durum wheat lines evaluated in 43 environments. The results of the multi-trait DLM were compared with univariate predictions of the GBLUP method and the univariate counterpart of the multi-trait DLM. The best predictions were observed in the absence of genotype × environment interaction term in the univariate and multivariate DLM. Overall, the deep DLM proved to be a practical approach for predicting traits in the context of genomic selection.

The remaining manuscripts addressed the genetic dissection of key traits for durum wheat yield and grain quality. Root system architecture features are increasingly investigated as proxy traits to optimize yield under drought conditions (Maccaferri et al., 2016; Bektas et al., 2020; Alemu et al., 2021). A GWA study allowed Alahmad et al. to identify a major quantitative trait locus (QTL) for seminal root angle on chromosome 6A harboring candidate genes related to gravitropism, polar growth, and hormonal signaling. The authors discussed the potential to deploy root architectural traits to enhance yield stability in environments with limited rainfall.

A more profitable and sustainable durum production scheme relies on novel cultivars that express enhanced resistance to Fusarium Head Blight (FHB) and the three rust diseases. Using a cytogenetic strategy, Kuzmanović et al. equipped durum wheat-*Thinopyron ponticum* recombinant lines with a *Thinopyrum elongatum* major QTL for FHB resistance. Then, a *Th. ponticum* 7el1L arm segment containing the exceptionally effective FHB resistance QTL from *Th. elongatum* together with *Lr19* (leaf rust resistance) and *Yp* (yellow pigment content), was also inserted onto 7DL of bread wheat lines. Chromosome engineering was also deployed by Othmeni et al. to introgress *Amblyopyrum muticum* and D-genome chromosome segments into durum wheat using pentaploid crosses. Results highlighted the importance of the parental genotype when attempting to transfer/develop introgressions into durum wheat from pentaploid crosses. Novel leaf rust QTLs were identified by Kthiri et al. based on mapping populations derived from crosses of resistant cultivars to Mexican races of *P. triticina*, with a susceptible line. Genetic analyses of host reactions suggested oligogenic control of resistance in all populations and identified seven QTLs physically anchored to the durum wheat reference sequence (cv. Svevo; Maccaferri et al., 2019). These QTLs and closely linked markers are useful resources for gene pyramiding and breeding for durable leaf rust resistance in durum wheat.

The search of novel alleles is also pursued through mutagenesis. Harrington et al. screened the cv. Kronos TILLinG population to identify a locus controlling a dominant, environmentally-dependent chlorosis phenotype. A segregating population was classified into discrete phenotypic groups and subjected to bulked-segregant analysis using exome capture followed by next-generation sequencing which highlighted the association on chromosome 3A of *Yellow Early Senescence 1* with the mutant phenotype. Coupling next*-*generation sequencing with phenotyping of large TILLinG collections allows high-throughput mutation discovery and selection by genotyping. Fruzangohar et al. mutagenized an advanced durum breeding line and performed short-read Illumina sequencing of the exome of 100 lines. An exome reference generated from Svevo and Kronos facilitated reads alignment from the mutants which produced a 484.4 Mbp exome reference. The authors also developed a user-friendly, searchable database and bioinformatic analysis pipeline that predicts zygosity of the mutations discovered and extracts flanking sequences for rapid marker development.

Among yield components, kernel size and shape are important parameters for wheat profitability. Based on data from three environments and 118 RILs from a cross of landrace Iran 249 with cultivar Zardak, Desiderio et al. identified 31 QTLs and 9 QTL interactions for kernel size, and 21 and 5 QTL interactions for kernel shape. Landrace Iran 249 contributed the beneficial allele for most QTLs for kernel shape suggesting its considerable potential for further yield improvement.

End-use quality traits are critical to the success of durum world in the production of pasta, couscous, and other related products. The carotenoid pigment content confers a bright yellow color to pasta and provides some antioxidant capacity. Colasuonno et al. reviewed the genetics of pigment accumulation in durum wheat grain. The major QTLs, accounting for up to 60% variation, were mapped on 7L homoeologous chromosome arms and were explained by allelic variations of the Phytoene Synthase (PSY) genes.

Seed storage proteins are crucial in determining end-use properties of wheat and its nutritional value. Giancaspro et al. reviewed the genetic variability for quantity and composition of grain protein content (GPC), together with grain yield/spike (GYS) and thousand-kernel weight (TKW) in a durum wheat population obtained from an interspecific cross between a common wheat accession and the durum cv. Saragolla. Three major QTLs were detected for both GPC and GYS while eight QTLs influenced TKW. QTLs for GYS, TKW, and GPC overlapped only marginally, with beneficial alleles contributed by both parents. Gluten strength is also key to determine pasta quality of durum wheat grain. Using 162 DH lines from Strongfield × Pelissier, Ruan et al. reported two major QTLs on chromosomes 1B and 1A explaining 25.4–40.1% and 13.7–18.7% of the gluten strength variability, respectively. These QTLs expressed consistently across environments are of great importance to maintain gluten strength of Canadian durum wheat to current market standards while selecting for other traits.

As compared to bread wheat, the comparatively more limited processing and food functionality of durum wheat relates to kernel texture (hardness) and gluten strength. Morris et al. addressed both traits using *ph1b*-mediated translocations from bread wheat. For kernel texture, ca. 28 Mbp of chromosome 5DS replaced about 20 Mbp of 5BS. Single Kernel Characterization System (SKCS) hardness was decreased from ca. 80 to 20 by the expressed puroindolines that softened the endosperm. Crosses with CIMMYT durum lines all produced soft kernel progeny, showing that soft durum can be considered a “tetraploid soft white spring wheat”; notably, excellent bread making potential was achieved by introgressing Dx2+Dy12 *Glu-D1* alleles in the Soft Svevo background.

In conclusion, facing and tackling the formidable challenges posed by the ongoing climate crisis will increasingly rely on faster genetic gains resulting from a systems-based approach integrating Triticeae multi-omics databases (Li et al., 2021), sequencing (Avni et al., 2017; Appels et al., 2018; Maccaferri et al., 2019; Brinton et al., 2020), breeder friendly phenotyping (Reynolds et al., 2020), deep learning (Wang et al., 2020) and modeling (Condon, 2020). The studies presented here underline the potential of leveraging durum wheat genomic resources (http://plants.ensembl.org/Triticum_turgidum/Info/Index; https://wheat.pw.usda.gov/GG3/node/759) and the tetraploid wheat diversity organized in the GDP and TGC panels (Figure 1; https://wheat.pw.usda.gov/GG3/content/november-2020-global-durum-genomic-resources-graingenes-0) as a genomic bridge with bread wheat (Maccaferri et al., 2015). The innovations offered by more sustainable wheat cultivars, once melded into resilient GxExM systems, will no doubt flourish (Beres et al.) for the benefit of farmers, consumers and the environment.

## Author Contributions

All authors listed have made a substantial, direct and intellectual contribution to the work, and approved it for publication.

## Conflict of Interest

The authors declare that the research was conducted in the absence of any commercial or financial relationships that could be construed as a potential conflict of interest.
